# Effects of *Pediococcus parvulus* 2.6 and its exopolysaccharide on plasma cholesterol levels and inflammatory markers in mice

**DOI:** 10.1186/2191-0855-2-66

**Published:** 2012-12-13

**Authors:** Cecilia Lindström, Olle Holst, Lars Nilsson, Rickard Öste, Kristina E Andersson

**Affiliations:** 1 Division of Biotechnology, Department of Chemistry, Lund University, Box 124, Lund, SE-221 00, Sweden; 2 Aventure AB, Scheelevägen 22, Box 719, Lund, SE-220 07, Sweden; 3 Department of Food Technology, Engineering and Nutrition, Lund University, Box 124, Lund, SE-221 00, Sweden; 4 Division of Applied Nutrition and Food Chemistry, Department of Food Technology, Engineering and Nutrition, Lund University, Box 124, Lund, SE-221 00, Sweden; 5 Department of Experimental Medical Science, Lund University, BMC D12, Lund, SE-221 84, Sweden

**Keywords:** *Pediococcus parvulus*, Cholesterol, Exopolysaccharide, Lactic acid bacteria

## Abstract

Intake of dietary fibres may reduce the prevalence of physiological risk factors of the metabolic syndrome, such as high plasma lipid levels and low-grade inflammatory state. Dietary fibres are usually of plant origin however microbial exopolysaccharides (EPSs) have analogue structures that could potentially exert similar physiological effects. *Pediococcus parvulus* 2.6 (Pd 2.6) excretes a ropy EPS and has previously shown probiotic potential. The aim of this work was to evaluate physiological effects of Pd 2.6 and its EPS *in vivo*. The live Pd 2.6 (both the ropy and non-ropy isogenic variant) and its purified EPS were fed to hypercholesterolemic LDL-receptor deficient mice for 6 weeks to investigate their effects on cholesterol levels and the inflammatory tone of the animals. Both variants of Pd 2.6 survived passage through the mouse gut fulfilling an important criterion of probiotics. The ability to produce EPS was conferring an advantage to survival (faecal recovery of 3.7 (1.9-8.7) vs. 0.21 (0.14-0.34) *10^8^ CFU, P < 0.001, median and 25th and 75th percentiles). The ropy Pd 2.6 decreased the levels of soluble vascular cell adhesion molecule-1 compared to the EPS alone (591 ± 14 vs. 646 ± 13 ng/ml, P < 0.05). An increase in liver weight in mice fed the purified EPS was observed, but with no change in liver lipids. No changes in blood lipids were detected in any group. Further the EPS induced growth of the caecal tissue and increased the amount of caecal content showing bulking properties like that of a dietary fibre.

## Introduction

Central obesity, hypertension, dyslipidaemia and insulin resistance are all independent risk factors for cardiovascular disease and type 2 diabetes. Co-occurrence of these metabolic abnormalities is stated as the metabolic syndrome (Eckel et al. 2010) which is associated with elevated levels of pro-inflammatory cytokines and inflammatory markers indicating a chronic inflammatory state (Galisteo et al. 2008). Life style intervention, including dietary changes, is suggested as a primary therapy for the metabolic syndrome (Grundy et al. 2005) and dietary fibres, including β-glucans, have been identified as important substances in the management of the metabolic abnormalities within the syndrome (Delzenne and Cani 2005; Galisteo et al. 2008). Recently the composition of the intestinal microflora has been acknowledged as an important factor in regulating the body weight (Bäckhed et al. 2004; Cani and Delzenne 2009; Ley et al. 2006) and could be used in fighting central adiposity. The microbiota may be altered by prebiotics that selectively stimulate the growth and/or activity of intestinal bacteria that can be associated with improvement of the host health. Such prebiotics should resist gastric acid and hydrolysis by host enzymes to be able to reach the gut and there be selectively fermented by the microbiota (Gibson et al. 2004). Another approach for modulation of physiological parameters is through the application of probiotics; living microorganisms which confer a health benefit to the host when administered in sufficient amounts (FAO/WHO 2002). The genus *Bifidobacterium* and species belonging to the group of lactic acid bacteria (LAB) like *Lactobacillus* are the most commonly used probiotics (Saad et al. 2013).

LAB may be isolated from different fermented foods such as dairy products, cereals, vegetables and various beverages. These strains are acid tolerant and may be adaptable to intestinal conditions and survive the passage through the gastrointestinal tract. This opens up the possibility to use potential probiotic strains from other niches than those of animal origin that are commonly used. The production of exopolysaccharides (EPSs) is a trait common to several LAB (Duboc and Mollet 2001) and there are numerous reasons for choosing EPS-producing LAB to test their potential probiotic properties. It has previously been shown that LAB and prebiotics show a synergistic effect on cholesterol metabolism (Mandal et al. 2009) and that LAB alone may provoke a cholesterol lowering effect *in vivo* (Bukowska et al. 1998; Park et al. 2007; Wang et al. 2009; Xie et al. 2011) where the presence of EPSs might increase the effect (Tok and Aslim 2010). The presence of EPSs from various LAB has been shown to reduce biofilm formation of pathogens *in vitro* (Kim et al. 2009), modulate adhesion to intestinal epithelial cells *in vitro* depending on type and dose of EPS (Ruas-Madiedo et al. 2006), reduce colitis in mice (Şengül et al. 2011) and selectively enhance the growth of bifidobacteria (Hongpattarakere et al. 2012; Korakli et al. 2002; Salazar et al. 2008; Salazar et al. 2009) showing a prebiotic potential. Further the EPS kefiran suppress increases in blood pressure, reduce serum cholesterol levels, lower blood glucose levels in rats (Maeda et al. 2004) and induce an immunological response in mice gut mucosa (Vinderola et al. 2006) indicating that EPS may be used to control the abnormalities occurring in the metabolic syndrome.

Since both LAB and their EPSs may have health beneficial effects it is of interest to compare physiological parameters after ingestion of pure EPS and the live bacteria. The LAB *Pediococcus parvulus* derived from alcoholic beverages has a great capacity to withstand environmental stress (Dols-Lafargue et al. 2008) and it has been shown to resist gastrointestinal stress *in vitro* (Fernández de Palencia et al. 2009; Immerstrand et al. 2010). *P. parvulus* 2.6 (Pd 2.6) has been isolated as a contaminant in Basque Country cider (Fernandez et al. 1995) and it produces a 2-substituted (1,3)-β-D-glucan (β-glucan) that is excreted into the surroundings as a ropy EPS causing unwanted alterations to the beverage known as ropiness; long slimy filaments of EPS may be picked up from the liquid (Duenas-Chasco et al. 1997). (Mårtensson et al. 2005) showed that a ropy oat based product fermented with Pd 2.6 induced a hypocholesterolemic effect in humans whereas the fermented non-ropy oat product did not (Martensson et al. 2005). Further, the ropy oat based product fed to rats in a freeze dried, sterilized form did not reduce plasma cholesterol; partly explained by the fact that the active component was given in a dry form compared to a fully solubilized form (Martensson et al. 2002). It should also be noted that the feed had been sterilized, hence no living Pd 2.6 was present in the product. This raises the question whether the cholesterol-lowering effect was attributed to the live Pd 2.6, the presence of EPS in the product or a synergistic effect with the oats. In addition the solubilisation of the material needs to be taken into account. The present investigation was set up to try to elucidate whether the hypocholesterolemic effect could be attributed to either the EPS or the live Pd 2.6. Mice were fed a diet containing purified EPS from Pd 2.6 (2%) and live Pd 2.6 both as the ropy and the isogenic non-ropy variant. By including the non-ropy variant synergistic effects between the bacteria and the EPS could be ruled out. The different additions were incorporated into the diets in a wet form. The survival of Pd 2.6 through the gastrointestinal passage and possible bioactive properties elicited by the Pd 2.6 and/or its EPS were evaluated. Specifically it was investigated if Pd 2.6 and/or its EPS have cholesterol-lowering activities or anti-inflammatory effects of low-grade systemic inflammation – both of importance in the metabolic syndrome.

## Materials and methods

### Production of EPS from ropy *Pediococcus parvulus* 2.6

#### Growth of Pd 2.6

Pd 2.6 was obtained from the Universidad de Pais Vasco culture collection, San Sebastian, Spain and stored at -80°C in MRS broth (Merck, Darmstadt, Germany) containing 20% glycerol (v/v). After revival the culture was routinely kept at 4°C in MRS broth containing 5% ethanol (Prolabo, VWR, Fontenay-sous-Bois, France), and subcultured every two weeks. Active liquid cultures were stepwise inoculated (2-5%) in MRS broth containing 0.2% sodium thioglycolate (Sigma, Sigma Aldrich Chemie, Steinheim, Germany) as oxygen scavenger. Finally 200 litres of media (Bioreactor BR600, Belach Bioteknik AB, Stockholm, Sweden) was inoculated and kept under soft agitation (20 rpm) to be able to maintain a constant temperature of 30°C. The head-space was flushed with nitrogen to keep an anaerobic atmosphere. The cultivation was not pH-controlled. The fermentation was terminated after 5.5 days and the broth was stored at 4°C until processed.

#### Isolation and purification of EPS

Trichloroacetic acid 40% (w/v) (Carl Roth, Germany) was added as 1/3 of the volume to the cultivation broth. The insoluble material was removed by centrifugation (7800 *g*, Sorvall RC 3C Plus, Thermo Scientific, 30 min room temperature). Three volumes of cold 95% ethanol (Solveco, Rosersberg, Sweden) were added to the supernatant and the precipitate was allowed to sediment at 10°C for at least 24 h. The precipitate was collected by centrifugation (7800 *g*) for 30 min at room temperature, sequentially dissolved in reversed osmosis water (Barnstead Nanopure, Thermo Scientific) and freeze dried (Ninolab, Upplands Väsby, Sweden). The freeze drier was programmed (Eurotherm, Malmö, Sweden) to start at -25°C for 3 h and then increase the temperature 1°C/min to -5°C and stay there for 12 h. The temperature was then increased to 5°C by 1°C/min and held for 12 h. Finally the temperature was increased 1°C/min to 25°C and held there for 1-2 days. The dry EPS was stored at room temperature.

#### Protein content of EPS

The protein content of the dry EPS was measured with the Pierce BCA Protein Assay Kit (Thermo Scientific, Rockford, IL, USA) according to the manual. The EPS was diluted in distilled water and analysed in duplicates.

### Molar mass determination of EPS using asymmetrical flow field-flow fractionation

#### Sample preparation

Samples for asymmetrical flow field-flow fractionation (AF4) were prepared by dissolving the EPS (1 mg/ml) in sodium nitrate buffer (10 mM) at room temperature by gentle stirring (200 rpm). The carrier liquid used in AF4 was an aqueous solution of 10 mM NaNO_3_ (AppliChem, Darmstadt, Germany) and 0.02% NaN_3_ (Kebo Lab, Spånga, Sweden).

#### Instrumentation

The system used was an Eclipse 3+ AF4 instrument connected to a Dawn Heleos II multiangle light scattering (MALS) detector and an Optilab T-rEX differential refractive index (RI) detector both operating at a wavelength of 658 nm (Wyatt Technology, Santa Barbara, CA, USA). The carrier flow was delivered by a pump with an in-line degasser; an auto-sampler handled the sample injection (1100 series, Agilent Technologies, Palo Alto, CA, USA). The AF4 Wyatt short channel was made up of a Wyatt wide spacer with a thickness of 250 μm, the tip to tip channel length 174 mm. A 20 nm pore size aluminium oxide filter in a Teflon filter holder was placed between the pump and the channel in order to ensure that a particle free solvent entered the channel. The ultrafiltration membrane on the accumulation wall was regenerated cellulose with a cut-off of 10 kDa (Microdyn Nadir Filtration, Wuppertal, Germany).

The sample was injected onto the channel at a flow rate of 0.2 ml/min for 1.0 min and the focusing flow rate was 1.0 ml/min. The injected amount was 400 μg. The cross-flow during elution started at 1.0 ml/min and was decreased exponentially with a half life of 4.0 min. The channel outlet flow was kept constant at 1.0 ml/min during the whole separation.

The light scattering data were processed using Astra software (Wyatt Technology). The molar mass was obtained by Berry’s method (Andersson et al. 2003; Berry 1966), fitting a straight line to the data obtained at scattering angles 38-99°. The refractive index increment value (dn/dc) was set to 0.146 ml/g based on the fact that most polysaccharides in aqueous solution have values in the range of 0.13-0.15 (Branderup and Immergut 1989). This may lead to an uncertainty of about 10% in the molar mass value obtained (Lambo-Fodje et al. 2007).

#### Plasmid curing

Polysaccharide synthesis in Pd 2.6 is controlled by a 567-amino acid, 65 kDa glucosyltransferase (GTF) that regulates polymerization of glucosyl residues from UDP glucose (Werning et al. 2006). The *gtf* gene is found on a 35-kb plasmid, pPP2 (GenBank accession no. AY999683). When the plasmid is cured the ropy trait is permanently lost and the strain becomes unable to synthesize EPS (Fernandez et al. 1995). In the present study the isogenic, non-ropy variant of Pd 2.6 was obtained by subculturing the ropy strain in MRS broth at 30°C leading to curing of its pPP2 plasmid and sequential permanent loss of its EPS producing trait. To confirm the new variant as Pd 2.6 16S rRNA gene sequencing was performed. Genomic DNA was extracted as described (Sambrook and W. Russell 2001) and the 16S rRNA coding gene was amplified by PCR using the universal primers 8 F (5^′^-AGA GTT TGA TCC TGG CTC AG-3^′^) and 1492R (5^′^-GGT TAC CTT GTT AGG ACTT-3^′^) (Eurofins, Germany) and Phusion High-Fidelity DNA Polymerase (Finnzymes, Vantaa, Finland). The PCR product was separated by agarose (0.8% w/v) gel electrophoresis, excised and purified with QIAquick PCR Purification Kit (Qiagen, Germany) according to the instructions provided by the manufacturer. Sequencing was performed by GATC Biotech AB (Konstanz, Germany) and the sequence was analysed with the Basic Local Alignment Search Tool (BLAST, http://www.ncbi.nml.nih.gov). Identification was performed on the basis of 16S rRNA sequence similarity.

#### Diets

The composition and energy content of the Western diets are presented in Tables 1 and 2 respectively. All diets were having microcrystalline cellulose (Avicel PH 101, FMC Biopolymer) as the fibre source. Fresh diets were prepared daily by adding MRS broth (Merck) to the pre-mixture (Research Diets Inc., New Brunswick, NJ, USA) at a concentration of 20% (v/w). The different groups were divided based on the additions made to the MRS broth. Group A served as control with pure MRS broth added. Purified EPS from Pd 2.6 was dissolved in MRS broth and added to the diet of Group B at a final concentration of 2% EPS based on dry matter. The amount of cellulose was decreased proportionally to give the same fibre content as the control (4%). For group C and D the MRS broth was inoculated with 0.6% Pd 2.6 of the ropy (Pd 2.6 R) and non-ropy (Pd 2.6 NR) variant respectively. The cultivations were incubated at 30°C for 48 h before addition to the diet. Consequently the diets of group C and D contained live bacteria upon administration. The amount of live bacteria administered was determined by plating serial dilutions of the MRS broth prepared in physiological saline on MRS agar (Merck) containing 5% ethanol (Solveco). The plates were incubated at 30°C for 48 h in anaerobic jars (Anaerocult, Merck).

**Table 1:** Formulation of the experimental diets (g/kg diet)

**Ingredient**	**Diets**	**EPS diet**
Casein, 80 mesh^a^	200	200
DL- methionine	3	3
Waxy corn starch	286	286
Maltodextrin 10	100	100
Sucrose	100	100
Cellulose	44	24
EPS	0	20
Butter, anhydrous^b^	200	200
Corn oil	10	10
Mineral mix S10026	10	10
Dicalcium phosphate	13	13
Calcium carbonate	5.5	5.5
Potassium citrate 1 H_2_O	16.5	16.5
Vitamin mix V10001	10	10
Choline bitartrate	2	2

*EPS*, exopolysaccharide.

^a^Casein is 88% protein.

^b^Anhydrous butter contains 230 mg cholesterol per 100 g. The amount of cholesterol in all diets were 0.46 g/kg diet.

**Table 2:** Energy and macronutrient content of the experimental diets

	**Diets**	**EPS diet**
Protein (% energy)	16	16
Carbohydrate (% energy)	48	48
Fat (% energy)	36	36
Energy (kJ/g diet)	16	16

*EPS*, exopolysaccharide.

#### Animals

LDL-receptor deficient mice (LDLr^-/-^) have successfully been used to study effects on blood lipids of dietary components (Andersson et al. 2010b; Dupasquier et al. 2007). The reduction of plasma cholesterol is relatively larger in LDLr^-/-^ compared to wild type mice (Andersson et al. 2010a; Andersson et al. 2010b) hence to be able to detect small changes in plasma cholesterol levels the LDLr^-/-^ was chosen for the present experiment. Female, homozygous LDL-receptor deficient mice (WEIBL. B6. 129S7 Ldlr^tm1Her^/J, Charles River, Sulzfeld, Germany) were randomly divided into four groups of ten animals upon arrival and acclimated for two weeks while being fed normal chow (R34 rodent chow, Lactamin, Vadstena, Sweden). The animals were housed in groups of 10 in plastic cages with housing material (22°C, relative air humidity 60%, 12 h light/dark cycle) having unlimited supply to feed and water. After acclimatization, at the age of 9-10 weeks (body weight 18.1 ± 1 g), the mice were fed a high fat, Western type experimental diet for six weeks. Feed intake and body weight were followed throughout the study.

At the end of the study the animals were anesthetized by isofloran and killed by cervical dislocation. Caecum content and livers were collected and snap frozen in liquid nitrogen and stored at -80°C. The emptied caecum tissue was weighed. Blood was collected from the heart and centrifuged (5000 *g*, Eppendorf) for 10 min at 4°C. The serum was stored at -80°C. The experiment followed national guidelines for the care and use of animals and were approved by Malmö/Lund ethical committee for laboratory animals (ethical permission number: M72-11).

#### Survival of Pd 2.6 through the gastro intestinal tract

Fresh faeces was collected from the mice and stored at -80°C until suspended in physiological saline and cultured on MRS agar plates containing 5% ethanol (Solveco). The plates were incubated aerobically at 30°C for four days.

#### Plasma cholesterol, triglycerides and serum alanine aminotransferase

Blood was collected from the saphenous vein into EDTA coated microvette tubes after 4 h of morning fasting and centrifuged (5000 *g*, Eppendorf) for 10 min at 4°C. The plasma was stored at -80°C. Plasma cholesterol and triglyceride concentrations were measured in duplicates using the Infinity Cholesterol/Triglyceride Liquid Stable Reagent (Thermo Scientific, Melbourne, Australia) according to the standard protocol. Alanine aminotransferase (ALT) was analysed in serum by the accredited laboratory Clinical Chemistry, Lund University Hospital, Lund, Sweden.

#### Total bile acids in faeces

At week 6 each group of animals were randomly divided into three subgroups of 3-4 mice and placed on grills for 24 h. Faeces were collected, lyophilized and mortared. Bile acids were extracted from faeces in 75% ethanol for two hours at 50°C while shaking (Yu et al. 2000). Solids were removed by centrifugation at room temperature. The supernatant was analysed in duplicates by the Colorimetric Total Bile Acids Assay Kit (Diazyme Laboratories, CA, USA) modified to suit a 96-well plate assay.

#### Lipid extraction from liver

Frozen livers were mortared under liquid nitrogen and freeze dried. Liver lipids were extracted in n-hexane:isopropanol (3:2, Merck) with 0.005% butylhydroxytoluene (Fluka) for 1 h at room temperature while shaking. The supernatant was removed after centrifugation and the pellet was washed three times in the solvent. The supernatants were collected and evaporated under nitrogen gas at room temperature. The lipids were redissolved in isopropanol containing 1% Triton X-100 (Sigma Aldrich) at 37°C while shaking for 15 min. Total cholesterol and triglycerides were analysed in duplicates using Infinity Cholesterol/Triglyceride Liquid Stable Reagent (Thermo Scientific, Melbourne, Australia) according to the standard protocol modified to suit a 96-well plate.

#### Markers of the inflammatory tone

Serum was thawed on ice and Serum amyloid A (SAA) and soluble vascular cell adhesion molecule-1 (sVCAM-1) were measured by ELISA kits (Tridelta Development, Kildare, Ireland and R&D systems, Abingdon, UK respectively) according to instructions provided by the manufacturers. Samples were analysed in duplicates.

#### Statistics

Data analysis was performed using Sigma Plot 11.0 (Systat Software Inc.) using one-way ANOVA for multiple comparisons followed by Tukey’s test to determine significance of difference between groups. Results are presented as mean values and their standard error of mean. Data failing the normality test were analysed by the Kruskal-Wallis one-way analysis followed by Dunn’s method for pair-wise multiple comparisons between groups, and expressed as median values with the 25th and 75th percentiles. Values with P < 0.05 were considered statistically significant.

## Results

### Production and characterization of EPS

**Figure 1:**
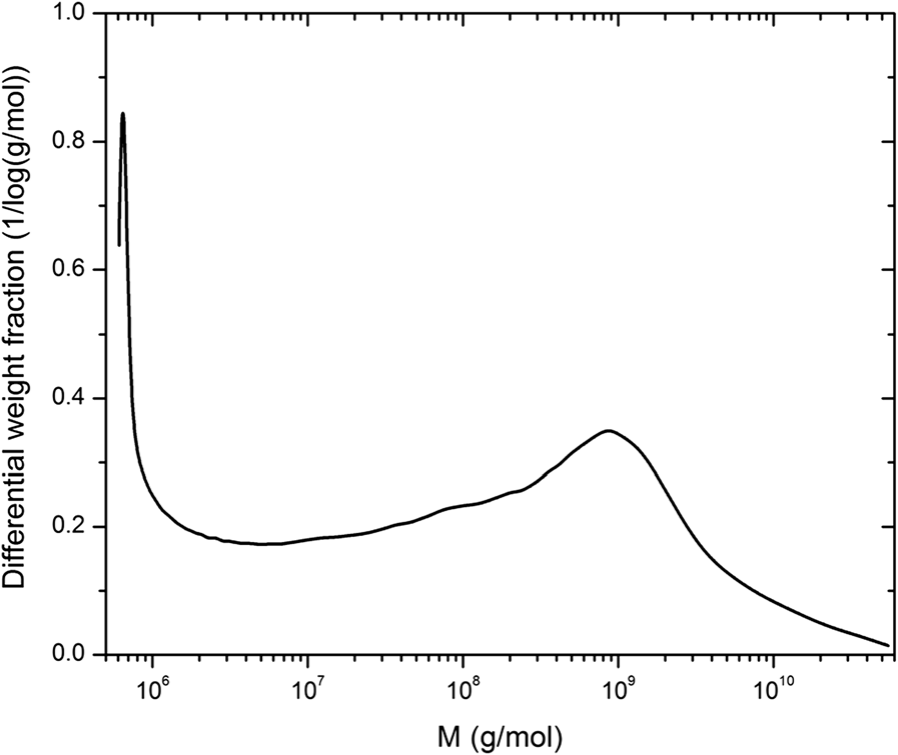
Differential molar mass distribution of the EPS produced by Pd 2.6 obtained from AF4-MALS-RI.

About 30 g of EPS was obtained from 100 litres of broth giving a yield of 300 mg/L. The EPS contained 10% protein. The main population of the EPS was showing a molecular weight of about 10^6^ Da (Figure 1).

### Survival of Pd 2.6 through the gastrointestinal tract

The groups fed live bacteria ingested Pd 2.6 (R and NR) at levels of 10^8^ CFU/g of feed (3.4*10^9^ CFU/mouse and day). In the present study faeces were inoculated on MRS containing ethanol (5%) to selectively grow Pd 2.6. Both the ropy and non-ropy variants were present as the only bacteria on the plates as confirmed by microscopy. The recovery of Pd 2.6 R was 17-fold higher than Pd 2.6 NR (Table 3).

**Table 3:** Pd 2.6 recovered in faeces

	**Pd 2.6 in faeces**		
		**(10**^**8**^ **CFU)**	
	**Median**	**25-75%**	***n***
Pd 2.6 R	3.7^a^	1.9-8.7	*12*
Pd 2.6 NR	0.21^b^	0.14-0.34	*10*

Normality test failed so a Mann-Whitney Rank Sum Test was performed. Data are expressed as median and 25th and 75th percentiles. Groups with unlike superscript letters were significantly different (P < 0.001).

### Physiological responses

There was no difference in feed intake (3.4 ± 0.04 g per mouse and day) and final body weight (23.6 ± 0.4 g) between groups as expected on an isocaloric diet. Caecal tissue weight and caecal content were significantly increased in the EPS group compared to the control (Table 4) showing a bulking effect resulting in enlargement of the organ. The faecal output and amount of total bile acids in faeces did not differ between groups (Table 4), and neither did plasma cholesterol and triglycerides (Table 5).

**Table 4:** Caecal tissue weight, caecal content, faecal output and concentration of bile acids in faeces

	**Caecal tissue weight**			**Caecal content**			**Faecal output**			Bile acids in faeces*		
		**(g)**			**(g)**		(g/mouse*24 h)			(μmol/mouse*24 h)		
	**Mean**	**SEM**	***n***	**Mean**	**SEM**	***n***	**Mean**	**SEM**	***n***	**Median**	**25-75%**	***n***
Control	0.047^a^	0.002	*10*	0.14^a^	0.01	*10*	0.124	0.026	*3*	1.5	1.2-1.8	*3*
Pd 2.6 EPS	0.057^b^	0.002	*10*	0.18^b^	0.01	*10*	0.128	0.029	*3*	1.3	1.0-1.9	*3*
Pd 2.6 R	0.048^a^	0.002	*10*	0.13^a^	0.01	*10*	0.123	0.017	*3*	1.9	1.4-2.0	*3*
Pd 2.6 NR	0.051^ab^	0.002	*10*	0.16^ab^	0.01	*10*	0.113	0.028	*3*	1.8	0.8-2.3	*3*

*n*, number of observations.

Statistics were calculated using one-way ANOVA for multiple comparisons. Tukey’s test was used for pairwise comparisons of means.

* These data failed the normality test so a Kruskal-Wallis one-way analysis of variance on the ranks was run followed by Dunn’s method for pairwise multiple comparisons. Groups with unlike superscript letters were significantly different (P < 0.05).

**Table 5:** Concentrations of plasma cholesterol, triglycerides and serum SAA and sVCAM-1

	Plasma cholesterol*			Plasma triglycerides*				SAA*			**sVCAM-1**	
	**(mmol/L)**			**(mmol/L)**				**(ug/ml)**			**(ng/ml)**	
	**Median**	**25-75%**	***n***	**Median**	**25-75%**	***n***	**Median**	**25-75%**	***n***	**Mean**	**SEM**	***n***
Control	23.9	20-25	*10*	4.6	4.4-5.8	*10*	25.8	22.2-27.6	*9*	610^ab^	12	*10*
Pd 2.6 EPS	24.8	22-26	*10*	5.5	4.6-6.7	*10*	24.9	24.0-31.1	*10*	646^a^	13	*10*
Pd 2.6 R	22.0	21-24	*10*	4.3	3.5-5.8	*10*	24.7	20.0-29.5	*9*	591^b^	14	*10*
Pd 2.6 NR	20.2	16-25	*10*	4.3	3.4-5.3	*10*	21.5	20.2-26.3	*10*	629^ab^	10	*10*

*SAA*, serum amyloid A; *sVCAM-1*, soluble vascular cell adhesion molecule-1.

Statistics were calculated using one-way ANOVA for multiple comparisons. Tukey’s test was used for pairwise comparisons of means.

* These data failed the normality test so a Kruskal-Wallis one-way analysis of variance on the ranks was run followed by Dunn’s method for pairwise multiple comparisons. Groups with unlike superscript letters were significantly different (P < 0.05).

Serum concentrations of inflammatory markers SAA and sVCAM-1 are presented in Table 5. The sVCAM-1 level was significantly lower for the group Pd 2.6 R compared to Pd 2.6 EPS.

The liver weight was significantly higher in the EPS group compared to the control. Sometimes this is a sign of fatty liver, but when the liver lipid contents were analysed no differences were found between groups (Table 6). Further the presence of elevated liver enzymes like ALT is used as an indicator of liver health however there were no significant differences between groups for serum ALT levels and there was no correlation between liver weight and serum ALT concentration (result not shown).

**Table 6:** Liver weight and concentration of total cholesterol and triglycerides in liver

	Liver weight per body weight*			**Total cholesterol in liver**			**Triglycerides in liver**		
		**(mg/g)**			**(mg/g)**			**(mg/g)**	
	**Median**	**25-75%**	***n***	**Mean**	**SEM**	***n***	**Mean**	**SEM**	***n***
Control	45.1^a^	44-47	*10*	13.5	0.45	*10*	60.3	5.0	*10*
Pd 2.6 EPS	58.1^b^	53-64	*10*	12.8	0.55	*10*	64.4	4.0	*10*
Pd 2.6 R	48.4^a^	44-51	*9*	11.9	0.30	*10*	50.3	4.5	*10*
Pd 2.6 NR	50.4^ab^	45-53	*8*	12.8	0.69	*10*	65.0	6.6	*8*

*n*, number of observations.

Statistics were calculated using one-way ANOVA for multiple comparisons. Tukey’s test was used for pairwise comparisons of means.

* These data failed the normality test so a Kruskal-Wallis one-way analysis of variance on the ranks was run followed by Dunn’s method for pairwise multiple comparisons.

## Discussion

Dietary fibres, specifically β-glucans, have been recognized as important substances with potential to regulate certain physiological parameters like the glycaemic response, hypertension and dyslipidaemia. There are several reports on the beneficial effects of cereal (Tiwari and Cummins 2011) and fungal (Kim et al. 2005; Mattila et al. 2000) β-glucans. Information about the effect of β-glucans from LAB is however scarce. Studies of specific physiological effects for structurally different β-glucans in animal and clinical trials are of great interest and it is of importance to characterize the β-glucans used (Chen and Raymond 2008). Therefore in the present study a β-glucan of known structure, the 2-substituted (1,3)-β-D-glucan produced by Pd 2.6 (Duenas-Chasco et al. 1997), was investigated concerning its effects on plasma lipids and inflammatory markers. The EPS was containing 10% protein of unknown identity however the protein content was not taken into account when the EPS was added to the diet. The molar mass distribution of the EPS was determined using AF4-MALS-RI (Figure 1) and the main population (highest peak) was showing a molar mass distribution of about 10^6^ Da in accordance with earlier reports (Lambo-Fodje et al. 2007). The population showing a higher molar mass (Figure 1) was of unknown origin however it could be an aggregated form of the EPS due to the high molar mass species present (M > 10^8^ g/mol). Figure 1 also shows the strength of AF4 being able to separate species of a very wide size range. The EPS was added to the experimental diet solubilised in its native form. The yield of the EPS is not feasible for commercial production of pure EPS therefore *in situ* production would probably be a better option. It has been shown that the presence of a food matrix like orange juice may increase the survival of Pd 2.6 when exposed to gastric stresses. The food is not negatively affected by the presence of Pd 2.6 which means that it can be used as a mean to administer the EPS and bacteria *in vivo* easily (Elizaquivel et al. 2011).

To our knowledge no reports exist on the survival of Pd 2.6 after passage through the gastrointestinal tract *in vivo*. In the present study both Pd 2.6 R and NR was isolated from faeces showing the ability of this LAB to survive the passage through the mouse gastrointestinal tract. The survival in the gut is a prerequisite for probiotic bacteria (FAO/WHO 2002) and the survivability of Pd 2.6 previously investigated *in vitro* (Fernández de Palencia et al. 2009; Immerstrand et al. 2010) was confirmed. The previous *in vitro* trial showed that the presence of EPS does not confer an advantage of survival for Pd 2.6 in the digestive tract (Fernández de Palencia et al. 2009) however when the EPS is heterologously expressed in *Lactobacillus paracasei* it gives an advantage of survival to both technological and gastrointestinal stresses compared to the wild type strain (Stack et al. 2010). The present study showed that the ropy variant was found in significantly larger numbers in faeces from mice compared to the non-ropy strain (Table 3). Since EPS production is the obvious thing that differs between the two isogenic variants it is suggested that the EPS increases the survival and ability to enumerate in the mouse gut *in vivo*. There are no defined dosages for probiotics today (Saad et al. 2013) however a daily dose of 3.4*10^9^ CFU for Pd 2.6 was sufficient for reaching and surviving in the intestinal tract of mice. Adhesion properties are often used as a selective factor when searching for new probiotics (Saad et al. 2013) and adhesion of Pd 2.6 to Caco-2 cells has previously been investigated *in vitro*. The results revealed that the ropy variant shows a higher level of adherence compared to the non-ropy variant (Fernández de Palencia et al. 2009). The ability of adhesion of Pd 2.6 R caused by the presence of EPS may explain the fact that it was found in higher numbers than Pd 2.6 NR in the gut of the mice.

The purified EPS induced growth of the caecum organ due to a bulking effect showing that it was acting as a dietary fibre. This effect of EPSs has been shown previously (Lindström et al. 2012). The bulking effect was not shown by Pd 2.6 R probably due to the concentration of EPS being too low to impart a significant effect. The bulking effect of polysaccharides is often caused by increased bacterial activity or increased water holding capacity. The faecal out-put and amount of water contained within the faeces (11-12.5%) was not different between groups indicating that bacterial activity was causing the bulking effect. The EPS from Pd 2.6 was probably acting as a substrate for various bacteria and further studies characterizing the microbiota would be interesting to perform trying to elucidate what kind of bacteria that were stimulated by the EPS. If these bacteria are belonging to the genera *Bifidobacterium* and/or *Lactobacillus* the EPS might be considered prebiotic.

The hypocholesterolemic effect seen in humans after ingestion of ropy fermented oats (Martensson et al. 2005) could not be reproduced in the present study where mice were fed single constituents from the complex fermented product. The pure EPS and live Pd 2.6 were hypothesised to be the active components. However these additions were not giving a cholesterol lowering effect in mice when administered as the sole ingredient. In the study of (Mårtensson et al. 2005) non-ropy fermented oats failed to show a cholesterol lowering effect. Thus it seems like the hypocholesterolemic effect was achieved as a protective and/or synergistic effect between oats and the EPS and/or Pd 2.6. Oats and barley contain (1,3)(1,4)-β-D-glucans (Webster and Wood 2011) whereas fungal β-glucans are (1,3) linked with branches at the (1,6) position (Chen and Raymond 2008). Thus the β-glucan produced by Pd 2.6 has a different chemical structure compared to those β-glucans that have previously been reported to exert hypocholesterolemic effects. Hence the lack of effect by the EPS may be explained by the absence of structural features that are needed for plasma lipid regulation.

(Notararigo et al. 2011) tested the influence of pure EPS from *Pediococcus* strains on gut mucosa *in vitro* by analysing the cytokine production by macrophages. They showed that the purified EPS increases the interleukin 10/tumour necrosis factor α ratio indicating an anti-inflammatory effect (Notararigo et al. 2011). In the present study the acute phase proteins SAA and the soluble variant of the adhesive molecule VCAM-1 were measured to investigate whether the EPS or live Pd 2.6 induced an anti-inflammatory response in mice. SAA did not differ between groups but sVCAM-1 was significantly lower for the Pd 2.6 R group compared to the pure EPS. There was a significant difference (P = 0.045, student’s t-test) between Pd 2.6 R and Pd 2.6 NR where intake of the ropy variant resulted in lower levels of sVCAM-1. In agreement with this (Fernàndez de Palencia et al. 2009) showed that the presence of EPS counteracts the pro-inflammatory response observed with Pd 2.6. In their study the amount of EPS was produced by the bacteria itself. (Notararigo et al. 2011) did not report the concentration of EPS used in their study however if small amounts were used the result from the two above mentioned studies may indicate that a level of EPS that would be produced by the bacterium is giving anti-inflammatory effects. However an increased level of EPS (2%) used in the present study was increasing the inflammatory tone upon ingestion.

In conclusion it seems like a better choice to incorporate the EPS-producing bacteria rather than the pure EPS in foods both from a technological and physiological standpoint. The yield of the EPS is low and apparently it does not give the favourable immunological response observed with the bacterium. Pd 2.6 was able to survive the passage through the intestinal tract of mice, where the ability to produce EPS conferred an advantage to survival in the gut, strengthening the assumption that probiotic bacteria may be found outside the commonly used genera of *Bifidobacterium* and *Lactobacillus*. However the physiological responses investigated here for Pd 2.6 (R and NR) were not different compared to the control group without bacteria and further studies are needed to determine specific positive physiological effects correlated to the presence of Pd 2.6 before it can be considered probiotic.

## Competing interest

RÖ has economical interest in Aventure AB that participated in the financing of the Antidiabetic Food Centre, which funded the study. CL, OH, LN and KEA declare that they have no competing interest.
